# Organic-modified ZnS nanoparticles as a high-performance lubricant additive†

**DOI:** 10.1039/d2ra07295e

**Published:** 2023-03-01

**Authors:** Chanaka Kumara, Beth Armstrong, Inwoong Lyo, Hong Wook Lee, Jun Qu

**Affiliations:** a Materials Science and Technology Division, Oak Ridge National Laboratory Oak Ridge TN 37830 USA kumarack@ornl.gov qujn@ornl.gov; b Institute of Advanced Technology Development, Hyundai Motor Corporation Republic of Korea

## Abstract

Lubricants are essential in transportation vehicles and industrial machinery to improve the lifetime of moving components. Antiwear additives in lubricants significantly minimize wear and material removal due to friction. While a wide range of modified and unmodified nanoparticles (NPs) have been extensively studied as lubricant additives, fully oil-miscible and oil-transparent NPs are essential to improve performance and oil visibility. Here, we report dodecanethiol-modified oil-suspendable and optical-transparent ZnS nanoparticles (NPs) with a nominal diameter of 4 nm as antiwear additives to a non-polar base oil. The ZnS NPs formed a transparent and long-term stable suspension in a synthetic polyalphaolefin (PAO) lubricating oil. The ZnS NPs in PAO oil at 0.5 or 1.0 wt% concentration demonstrated excellent friction and wear protection. The synthesized ZnS NPs showed 98% wear reduction compared to the neat PAO4 base oil. For the first time, this report showed the outstanding tribological performance of the ZnS NPs benchmarked to the commercial antiwear additive zinc dialkyldithiophosphate (ZDDP) with an additional 40–70% wear reduction. Surface characterization revealed a ZnS-derived self-healing polycrystalline tribofilm (<250 nm), which is key to superior lubricating performance. Our results indicate the potential of ZnS NPs as a high-performance and competitive antiwear additive to ZDDP, which has broad transportation and industrial applications.

## Abstract 2

Oil-miscible and oil-transparent organic modified ZnS nanoparticles were developed as an antiwear additive to improve lubricant performance.

## Graphical abstract

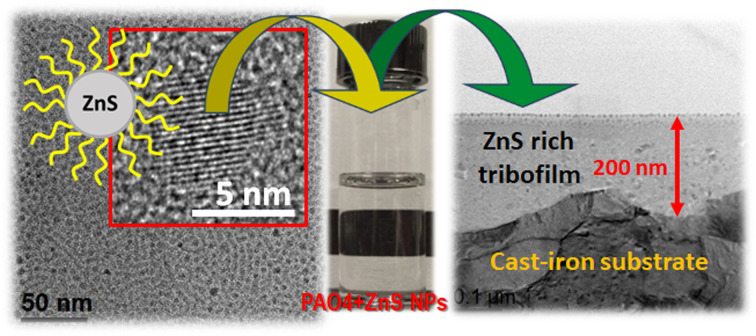

## 1. Introduction

Nanoparticles (NPs) have attracted significant attention in a wide range of fundamental and applied research due to their unique physical and chemical properties. In lubrication science, NPs are used as protective coatings, solid lubricants, and lubricant additives. Metallic, ceramic, and carbon-based nanomaterials are widely used as oil additives.^1–9^ Good dispersibility and stability in the oil are often required to achieve desired functionality and performance. The interaction between NPs and oil molecules could be improved either by adding dispersants or by NP surface modification. The addition of dispersant provides favorable interface to interact NPs with oil molecules by dipole–dipole or van der Waals interactions. Also, surfactant passivation could bring additional stability to the NPs by minimizing surface energy. However, variation in local temperature, pressure, and density might alter the dispersant activity leading to poor NPs miscibility and aggregation in the oil. However, surface modification of NPs has advantages of intact surface interaction with oil molecules and therefore bear minimal influence from environmental fluctuations. In early reports, we had successfully developed organic-modified metallic Ag^10^ and Pd NPs,^11^ which form stable suspensions in a polyalphaolefin (PAO) lubricating oil to effectively reduce friction and wear. However, the optical absorption of the Ag and Pd NPs additives alters the oil opacity to intense brown or dark red color, making them less appealing in practical use, despite their superior antiwear performance.

Semiconductor nanomaterials possess unique size-dependent optical and electronic properties. Groups II–VI semiconductors include periodic table elements combination of groups II and VI such as CdSe, CdS, ZnSe, and ZnS. These materials display a quantum-confined effect due to the arrangement of band structure into discrete quantum levels resulting from nanoscale size. Zinc sulfide is one of the most important groups II–VI semiconductors due to its wide bandgap (3.6 eV), low cost, and non-toxic nature.^12^ Moreover, ZnS NPs show optical transparency over a wide energy range, which has promising application in transmission windows in solar cells, liquid crystal displays, and light-emitting diodes.

Zinc dialkyldithiophosphates (ZDDPs) are the most common antiwear additives used in engine oils but they are under pressure to be removed because their organophosphate anions are known to poison the exhaust emission control catalysts by forming a glassy phosphate layer on top of them.^13,14^ Therefore, the tribo-active and phosphorus-free ZnS NPs would be an alternative lubricant additive if they could be stably suspended in oil. Some of the previous oil-suspendable ZnS NPs were based on organophosphate surface modification, such as Cyanex 302 [di-(2,4,4-trimethylpentyl)monothiophosphinic acid],^15^ dithiophosphate,^16^ or di-*n*-hexadecyldithiophosphate (DDP). Liu *et al.* performed the tribological tests using non-coated ZnS NPs and DDP-coated ZnS NPs. While DDP coated ZnS NPs performed well in tribological tests, non-coated ZnS experienced solubility issues with oils.^17^ Although organophosphate surface modifications gain an advantage from phosphorus, the performance of ZnS NPs might be obscured by such a surface modification. On the other hand, the organophosphate ligand would impair the exhaust emission catalysts. Therefore, selections of NPs and surface modification are equally important in the development of NPs for tribological applications.

**Fig. 1:**
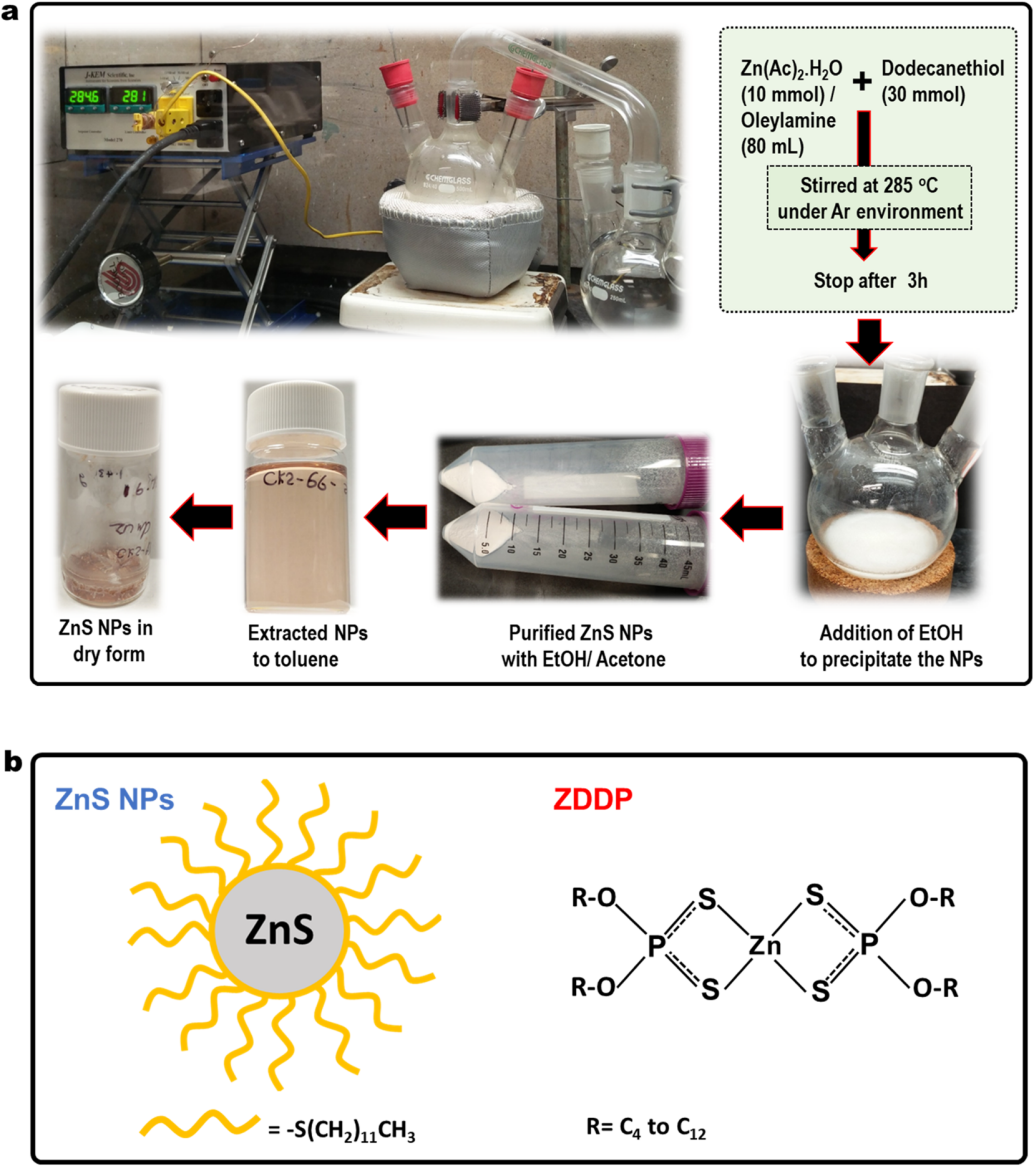
(a) Pictures showing overall ZnS nanoparticle synthesis process, including setup, conditions, and processing. (b) Model structures of the ZnS NPs and ZDDP. The R represents an alkyl group in ZDDPs.

In this report, we synthesized oil-suspendable and optical-transparent ZnS nanocrystals of ∼4 nm diameter as a candidate antiwear additive and dodecanethiol for organic surface modification as shown in Fig. 1a. The ZnS NPs were characterized using Transmission Electron Microscopy (TEM), UV-visible spectroscopy, and thermogravimetric analysis (TGA). Tribological bench tests were conducted to investigate the friction and wear performance of ZnS NPs benchmarked against a commercial ZDDP. The model structure of ZnS NPs and ZDDP are illustrated in Fig. 1b. The direct comparison of the antiwear performance to the commercial ZDDP was lack in most nanoparticle-based literature reports, but necessary to recognize competitive antiwear performance with the high-performance market products. The synthesized ZnS NPs showed 98% wear reduction compared to the neat PAO4 base oil. For the first time, this report show the extraordinary tribological performance of the ZnS NPs, which outperformed a widely use zinc dialkyldithiophosphates (ZDDP) antiwear additive by 40–70% wear reduction.

## 2. Experimental section

### 2.1 ZnS nanoparticles synthesis

Synthesis of dodecanethiol functionalized ZnS NPs was carried out using a method similar to the literature procedure with modification as shown in Fig. 1a.^18^ In brief, zinc acetate (Zn(Ac)_2_,10 mmol) and oleylamine (80 mL) were mixed in three-neck round bottom flask and temperature raised to 100 °C under continuous stirring until metal complexes were completely dissolved in the solvent. Then dodecanethiol (30 mmol) was added to the solution, and the temperature was raised to 285 °C and maintained at that temperature for 3 hours to complete the reaction. The reaction was done in an argon environment. After cooling down to room temperature, EtOH was added to precipitate the NPs. Then, further purification was carried out with EtOH/acetone mixture (1 : 1 by volume) to remove excess solvents and other byproducts. Finally, ZnS nanoparticle was extracted to toluene solution. Then, the mixture was vacuum dried to concentrate the ZnS NPs. The ZnS NPs exhibit light pink color in both solid and liquid forms. The chemical structures of the ZnS NPs, ZDDP and their abbreviation are presented in Fig. 1b. ZnS nanoparticles mainly consist with Zn and S in 1 : 1 atomic ratio. The co-ordination geometry at Zn and S is tetrahedral while the oxidation states are Zn^2+^ and S^2−^. The outer sulfide layer attached to the organic ligand is –C_12_H_25_ in this case. Simulation studied shows existence of three regions in the relaxed configurations (a) a 4-coordinated crystalline core at which Zn and S atoms keep their initial tetrahedral arrangement, (b) a distorted network of 4-coordinated atoms surrounding the crystalline core and (c) the surface structure which consists of a network of 3-coordinated atoms.^19^ Outer organic layer consists with C_12_H_25_– hydrocarbon chain that responsible for NPs suspensibility in oil.

The ZDDP molecules contains Zn, S, P, O, C and H, and two dithiol phosphate molecules chelating to the Zn atom. The atomic ratio of Zn, P, S in ZDDP molecules is 1 : 2 : 4. The R group represent alkyl hydrocarbon chain typically vary from C_4_ to C_12_ and it allow molecules to dissolve in oil.

As a antiwear additive ZDDP reacts under boundary/mixed lubrication condition to form protective zinc, sulfur and phosphate derived tribofilm. The thermal degradation of ZDDP in solution dependent on the alkyl groups and typically occurs between 130 and 230 °C. The thermal degradation route leads to a zinc phosphate, alkyl sulphides, thiols, and olefins.^13^ The ZDDP synthesis is two-step process that involved working with combustible solids that require a moisture free environment and stepwise purification process. In contrast, ZnS NPs synthesis is one-pot synthesis at ambient environment. Therefore, ZnS NPs preparation would have less environmental impact compared with the ZDDP in manufacturing.

### 2.2 ZnS NPs' oil suspendability

**Fig. 2:**
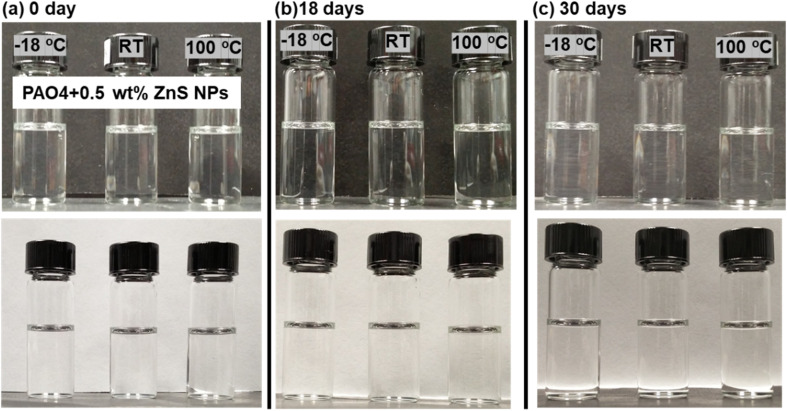
Pictures showing PAO4 + 0.5 wt% ZnS NPs (a) initial state and after being kept at −18 °C, RT and 100 °C for (b) 18 days and (c) 30 days. Black and white backgrounds were used to visualize the oil transparency.

Fully dried ZnS NPs exhibit a light pink color in its solid and solution form. The ZnS NPs seemed to be well dispersed in organic solvents and the PAO4 base oil to produce a transparent suspension. The addition of 1 wt% of NPs to the PAO4 oil changed the suspension color slightly pink but no noticeable color change for the concentration of 0.5% or below. The ZnS NPs stability and suspensibility in PAO4 were further examined as functions of time and temperature (at −18, 23 and 100 °C) as shown in Fig. 2. The exceptional stability of the organic modified ZnS NPs in oil was evidenced by an insignificant change in oil transparency or absence of visible precipitation after more than two years. The viscosities of the PAO4 base oil without and with ZnS NPs at three different temperatures are compared in Table 1. Evidently, the addition of NPs did not alter the oil viscosity.

**Table 1:** Oil viscosity of the PAO4 base oil and ZnS added oil

Lubricant	Viscosity (cP)		
	Temperature (°C)		
	23	40	100
Neat PAO4	29.7	15.2	3.2
PAO4 + 0.5% ZnS NPs	29.7	15.1	3.2

### 2.3 Tribological bench test

Boundary lubrication tests were conducted to examine the lubricant properties of ZnS NPs in the PAO4 oil, which were benchmarked against a secondary ZDDP (abbreviated as ZDDP). The ZnS NPs additive concentrations vary from 0.25 to 1.00 wt% in PAO base oil whereas ZDDP concentration maintain at 0.80 wt%. The addition of antiwear additive, 0.80 wt% ZDDP, to PAO base oil was to compliance with the upper phosphorus limit (800 ppm) defined by the International Lubricant Specification Advisory Committee (ILSAC) GF-6 specifications.

The tribological tests were conducted on a Plint TE-77 tribotester using a ball-on-flat reciprocating sliding configuration. A Grade 25 hardened (HRC 60) AISI 52100 steel bearing ball (10 mm dia.) was slide against a CL35 gray cast iron flat. The arithmetic average roughness (*R*_a_) of the ball was in the range of 25–50 nm based on the Grade 25 bearing ball specifications. The flat surface was polished using SiC abrasive papers to reach the roughness (*R*_a_) of ∼60 nm. The Vickers hardness was 878 and 245 HV at 500 gf micro-indentation for the steel ball and cast-iron flat, respectively. This material pair was used to simulate the common sliding interface for a steel piston ring against a gray cast iron cylinder wall in an actual engine. The tests were carried out at 23, 100 or 150 °C under a load of 100 N and an oscillation frequency of 10 Hz with a stroke of 10 mm for 1000 m.

The calculated Hertzian contact stress at the ball–flat interface at the beginning of the test was about 1.7 GPa. This is higher than the compressive yield strength of gray cast iron (∼800 MPa) and thus the cast iron flat surface is expected to yield upon contact until the actual contact pressure dropped to around 800 MPa. The contact pressure would further decrease along with the enlarged contact area during the wear test. Using the Hamrock–Dowson formula,^20^ the calculated central lubricant film thickness *t* at the beginning of the test is less than 15 nm. The composite roughness 
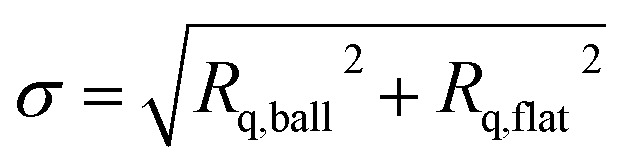
 was calculated to be above 100 nm. Therefore, the lambda ratio *λ* = *t*/*σ* is <1, indicating that the lubrication regime in the tribo-test was boundary lubrication.^12^

Friction force was captured *in situ* using a piezoelectric load cell. Wear volumes of both the ball and flat were quantified using a 3D optical profiler (Wyko NT9100 white light interferometer) aided by a Vision (version 4.10) software. Cylindrical and spherical curvature fittings were applied to flat and ball surfaces in wear volume calculations, respectively.

### 2.4 Characterization

#### Nanoparticle characterization

TEM grid containing NPs was prepared by drop-casting a toluene solution of ZnS NPs onto a carbon film supported on Cu grids. The NPs were examined using a Hitachi HF-3300 transmission electron microscope at 300 kV. Images were analyzed using ImageJ (version 1.46r) software. The particle size distributions of the ZnS NPs in PAO4 were measured by using a NanoBrook 90Plus PALS dynamic light scattering (DLS) instrument. The PAO4 + 0.5 wt% ZnS NPs and PAO4 + 2 wt% ZnS NPs suspension were prepared for the measurement. Optical spectra of the ZnS NPs were measured in a hexane solution using a Varian Cary 5000 spectrophotometer at the 250–800 nm range. Thermogravimetric analysis (TGA) of organic modified ZnS NPs was performed with an AutoTGA-2950 (TA Instruments) under an N_2_ flow at a 10 °C min^−1^ heating rate.

#### Tribofilm characterization

A Hitachi S4800 scanning electron microscope (SEM) equipped with energy-dispersive spectroscopy (EDS) was used for the wear scar morphology and relative element composition analysis. A focused ion beam (FIB) was used to lift-out thin cross-sections of the wear scar using a Hitachi NB5000 FIB equipped with a gallium ion source. A thin carbon layer was deposited on the wear scar before starting the FIB process to protect the tribofilm. Tribofilm cross-section were analyzed using a JEOL JEM 2200-AC Scanning Electron Microscopy (STEM) equipped with a Bruker-AXS Silicon Drift EDS detector system at 200 kV. The relative element composition of the tribofilm was determined using standardless semi-quantitative analysis routines in the Bruker Esprit (version 2) software.

## 3. Results and discussion

### 3.1 Synthesis and characterization of ZnS NPs

Synthesis of oil-suspendable ZnS required a long-chain carbon backbone to improve the intermolecular interaction with the non-polar PAO oil. Thus, dodecanethiol (C_12_H_25_SH with SC_12_ backbone) was selected to modify the ZnS NPs surface. The function of dodecane thiol in this synthesis has several advantages in addition to acting as a sulfur source to produce ZnS, when compared to the use of elemental sulfur: (a) thiol group attached to the long hydrocarbon chain at the outer-shell act as a protective layer preventing NPs aggregation (b) organic nature of the hydrocarbon chain facilitate ZnS suspensibility in oil by van der Waals interactions. Final synthesis process was optimized with Zn(Ac)_2_ precursor to produce the gram quantities of ZnS NPs. Oleylamine was used as a solvent in this non-aqueous synthesis route.

ZnS NPs formation proceeds *via* the formation and decomposition of Zn_*x*_(SC_12_H_25_)_*y*_ organo-transition metal complexes. At a reaction temperature of ∼285 °C, a portion of dodecanethiol subjected thermolysis producing S^2−^ ions which then react with Zn_*x*_(SC_12_H_25_)_*y*_ complexes to form Zn_*x*_S_*z*_(SC_12_H_25_)_*z*_ clusters. The Zn–S bond formation produced ZnS core at the center and S–C bonding produced a protective inorganic–organic interface. Oleylamine plays an important role not only by acting as a solvent but also by slowly releasing the adsorbed Zn to the Zn_*x*_S_*z*_(SC_12_H_25_)_*y*_ clusters to form monodisperse ZnS NPs. The Na_2_S is another frequently used sulfur source for ZnS NPs synthesis while introducing surface modification group to minimize the surface energy.^16,21^ However, such a protocol might be suitable to produce ZnS NPs for polar oils such as PEG 400 or liquid paraffin.

**Fig. 3:**
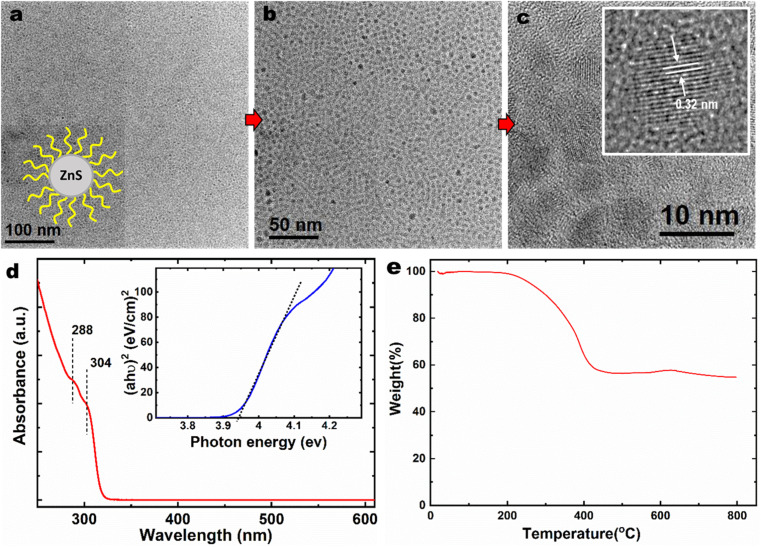
(a)–(c) TEM image of dodecane thiol modified ZnS NPs at different magnifications showing the model structure, particle shape, size, and size distribution. (d) UV-visible spectra of the ZnS NPs in hexane solution. Inset shows Tauc plot. (e) TGA analysis of the ZnS NPs in N_2_ environment.

Fig. 3a and b shows the TEM images of the ZnS NPs showing thousands of particles in low magnification images. Fig. 3a inset shows the model structure of the ZnS nanoparticles with the organic ligand attached. The NPs seem to arrange as a self-assembly layer on the TEM grid, and individual particles were well separated without NPs aggregation. The shape of particles is likely pseudohexagonal or pseudocubical with an average diameter of ∼4 nm. ZnS NPs lattice fringes indicate the interplanar spacing of 0.32 nm as shown in Fig. 3c inset.

The UV-visible spectra of the ZnS NPs in Fig. 3d show the absorption on-set at 320 nm. The bulk ZnS shows the absorption on-set at 340 nm.^22,23^ The ZnS NPs did not respond to the visible light due to their wide-bandgap. This blue-shifting of the absorption band (by 20 nm) indicates the quantum confinement effect of the synthesized ZnS NPs. The bandgap energy of ZnS NPs was determined using the Tauc plot^24^ to be 3.9 eV.

Thermogravimetric analyses were used to determine the organic content of ZnS NPs due to their hybrid organic–inorganic nature. Fig. 3e presents the TGA of the ZnS NPs acquired by ramping the temperature from 20 to 800 °C at a heating rate of 10 °C min^−1^ under N_2_ atmosphere to avoid possible ZnS oxidation. The organic content of the NPs was found to be 40 wt%. The initial weight loss of NPs started at 200 °C is due to the removal of the protecting group (boiling point of dodecanethiol is 275 °C) and weight changing continuously until 450 °C before reaching constant weight at 55 wt% due to the residual ZnS core. In contrast, many commercial additives, such as ZDDP, start to decompose at ∼200 °C and accompanied steep weight loss at around 250 °C.^25^ Dynamic light scattering technique was employed to determine the average particle size of ZnS NPs in PAO4 base oil. Two concentrations were used: ZnS NPs at 0.5 wt%, and 2 wt% in PAO4 and the particle sizes were found to be 10 ± 1 nm and 14 ± 2 nm, respectively. The PAO4 + 2 wt% ZnS NPs allows us to understand the ZnS NPs behavior in oil at high concentration. Still, ZnS NPs seem to have narrow size distribution in PAO4 (even at four times higher concentration) as a results of organic surface modification. The primary particle size of the ZnS NPs was measured to be ∼4 nm by TEM. Therefore, ZnS NPs seem to exist in small clusters in PAO4 oil due to attractive van der Waals interactions exert from organic ligand. Most importantly, the dodecanethiol organic modification seems minimize surface charge of naked ZnS NPs and prevent larger aggregation, agglomerations and precipitation. The aggregation occurred whenever the Brownian motion and attractive forces of the nanoparticles were more significant than the repulsive forces.^26^ On the other hand, the TEM size measurement did not account for the organic ligand thickness due to the low contrast from hydrocarbons, but DLS is accounts for scattering from the organic ligands, and, therefore, typically, DLS is expected to report larger particle sizes than TEM.

### 3.2 Tribological performance of ZnS NPs in oils

**Fig. 4:**
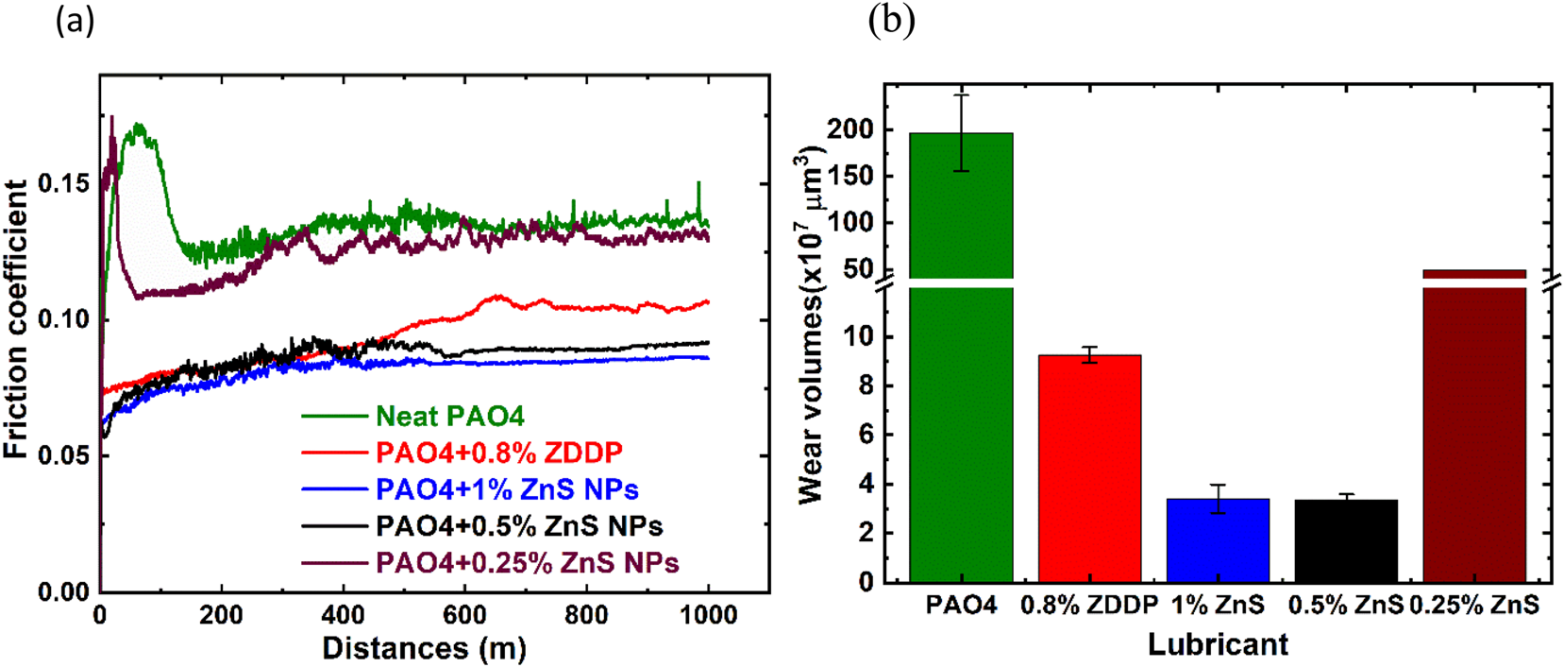
Friction (a) and wear (b) performance of PAO4 and ZDDP compared to the ZnS NPs at 100 °C.

The friction and wear performance of the ZnS NPs in PAO4 were first evaluated at 100 °C. Initial tests were conducted to examine the impact of the ZnS NPs concentrations as shown in Fig. 4 and Table 2. For the neat PAO4, a frictional spike was observed at the beginning of the sliding due to the lack of antiwear additive but recovered during the running-in process and reached a steady-state coefficient of friction (COF) ∼ 0.13. The COF of the PAO4 containing ZDDP (0.80 wt%) started at 0.075 and gradually increased with sliding distances and seemed to be reached steady-state COF around ∼0.10. Three different ZnS NPs additive concentrations were tested with PAO4 base oil. The addition of 1.00 or 0.50 wt% ZnS NPs into PAO4 base oil had more than 13–18% friction reduction compared to the ZDDP. However, the addition of 0.25 wt% of ZnS NPs appeared insufficient to protect the system with the friction behavior similar to the neat PAO4.

**Table 2:** Wear performance of PAO4, ZDDP, and ZnS NPs at the different concentrations at 100 °C

Lubricant	Wear volume (×10^7^ μm^3^)			Steady-state COF^a^
	Flat	Ball	Total	
Neat PAO4	196 ± 40.50	0.75 ± 0.06	196.75 ± 40.50	0.136
PAO4 + 0.8% ZDDP	9.24 ± 0.32	0.05 ± 0.01	9.27 ± 0.32	0.104
PAO4 + 1% ZnS NPs	3.33 ± 0.58	0.08 ± 0.01	3.41 ± 0.58	0.085
PAO4 + 0.50% ZnS NPs	3.20 ± 0.23	0.16 ± 0.10	3.36 ± 0.23	0.090
PAO4 + 0.25% ZnS NPs	48.60	0.86	49.46	0.130

a Steady-state COF determined by averaging the COF data of last 200 m.

As a widely used antiwear additive, the ZDDP performed well with more than 95% wear reduction than the neat PAO4 base oil. Interestingly, the addition of 0.50 wt% of ZnS NPs to the PAO4 base oil demonstrated superior wear protection than the ZDDP with additional 63% wear reduction. The addition of 1 and 0.50 wt% ZnS NPs had similarly wear performance, but 0.25 wt% was not enough to provide good wear protection. Therefore, the ZnS NPs concentration was determined to be 0.5 wt% or above for the PAO4 oil system. The focus of Fig. 4 tribological testing is to investigate the optimal ZnS NPs additive concentration. Since the antiwear performance of either 0.5 wt% or 1 wt% is significantly closer, we could expect that the PAO + 0.8 wt% ZnS NPs would have a similar performance which is still better than the performance of PAO + 0.8 wt% ZDDP. On the other hand, use of 0.80 wt% ZDDP is to maintain the upper phosphorus limit (at 800 ppm) according to the ILSAC GF-6 specifications. The ZDDP provide three tribologically active elements, Zn, S, and P (P is well known as the most triboactive element involved in tribofilm formation by providing phosphate compounds) compared to the ZnS NPs, which have only two (Zn and S) tribologically active elements.

**Fig. 5:**
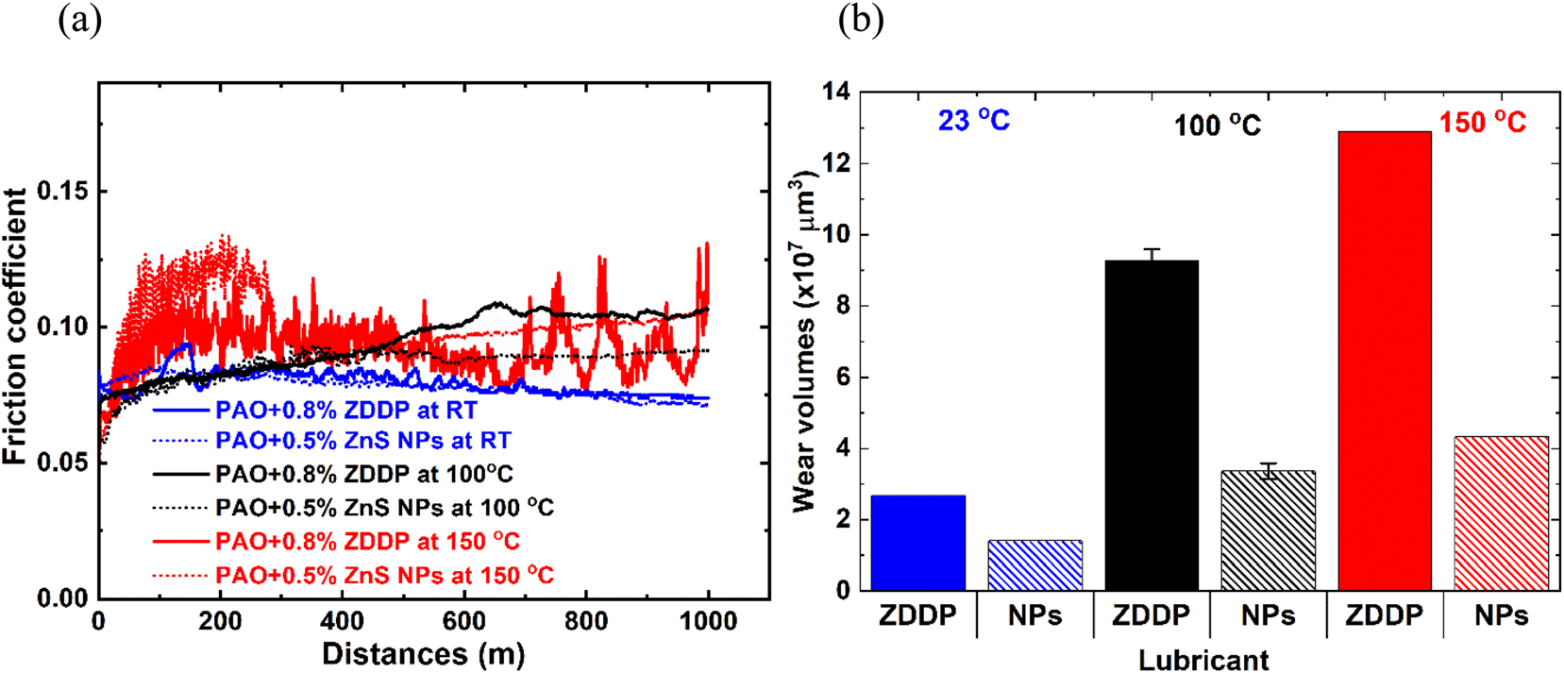
Friction (a) and wear (b) performance of ZDDP and ZnS NPs at different temperatures, RT (23 °C), 100, and 150 °C.

After determining the effective ZnS NPs additive concentration, further tribological tests were conducted to investigate the lubricant performance at different temperatures. The friction and wear performance of the ZDDP and ZnS NPs at RT, 100, and 150 °C are presented in Fig. 5 and Table 3. The ZnS NPs and ZDDP had similar friction traces at RT, but ZnS NPs produced lower friction at 100 °C. At 150 °C, ZDDP experienced unstable COF with several friction spicks over the 1000 m sliding. Although the ZnS NPs containing oil also experienced an initial frictional spick, the COF dropped and stabilized after 300 m sliding and reached to ∼0.10. Wear scar volumes were increased as temperature increased for both oil additives. ZnS NPs outperform the ZDDP at all three temperatures tested, with a 47% wear reduction at RT and 67% wear reduction in both 100 and 150 °C (Table 3).

**Table 3:** Friction and wear performance of ZDDP and ZnS NPs lubricated oil at different temperatures

Temperature (°C)	Lubricant	Wear volume (×10^7^ μm^3^)			Average COF^a^
		Flat	Ball	Total	
25	PAO4 + 0.8% ZDDP	2.67	0.002	2.672	0.075
	PAO4 + 0.5% ZnS NPs	1.40	0.005	1.405	0.073
100	PAO4 + 0.8% ZDDP	9.24 ± 0.32	0.05 ± 0.01	9.27 ± 0.32	0.104
	PAO4 + 0.5% ZnS NPs	3.20 ± 0.23	0.16 ± 0.10	3.36 ± 0.23	0.090
150	PAO4 + 0.8% ZDDP	12.90	0.05	12.90	0.091 ± 0.10
	PAO4 + 0.5% ZnS NPs	4.22	0.12	4.34	0.103

a Average COF determined by averaging the COF data of last 200 m.

### 3.3 Tribofilm analysis

**Fig. 6:**
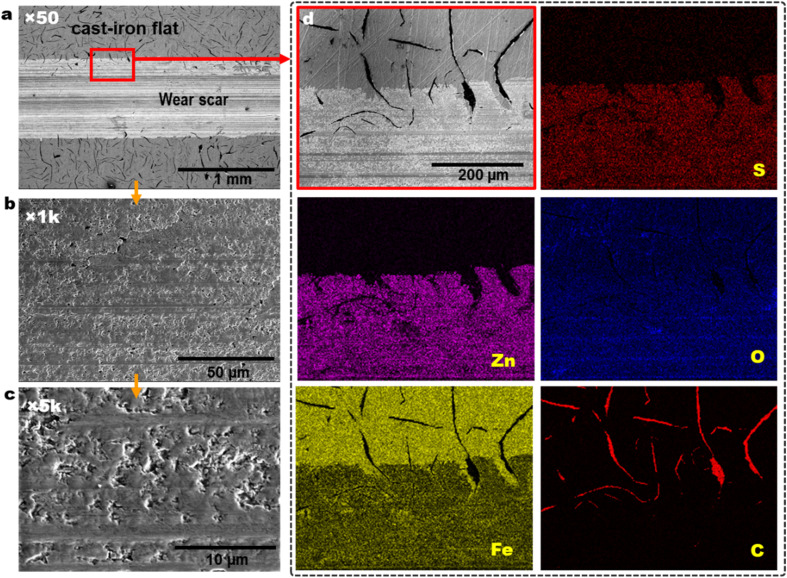
SEM wear scar morphology (a–c) and EDS elemental maps (d) of the cast-iron flat lubricated by PAO4 + 0.5 wt% ZnS NPs at 100 °C.

Fig. 6 shows the SEM wear scar morphology, and EDS element maps of the worn cast iron flat lubricated by PAO4 + 0.5 wt% ZnS NPs. Graphite flakes distribution on the cast iron surface is visible at outside the wear scar as black color strips in Fig. 6a. Wear track and pad-like ZnS tribofilm morphology could be seen from low magnification to high magnification SEM images of Fig. 6a–c. The EDS elemental maps show the co-distribution of Zn and S on top of the wear track in Fig. 6d, suggesting the tribofilm was derived from the ZnS NPs.

**Fig. 7:**
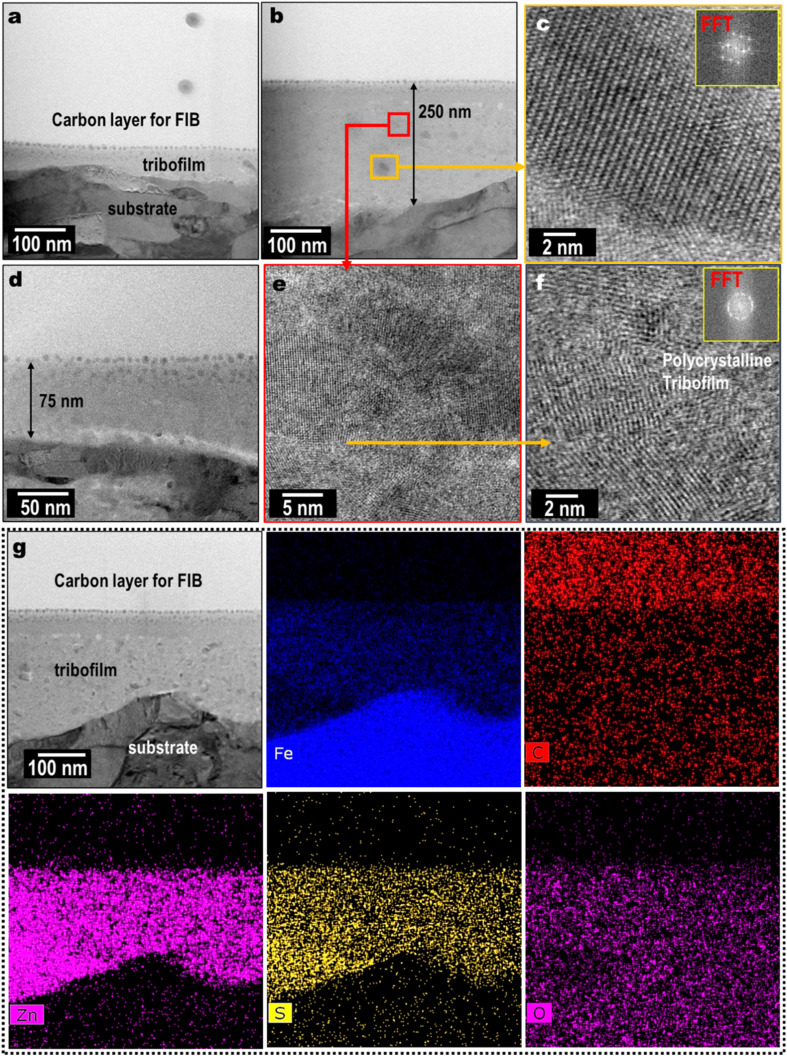
Cross-sectional STEM morphological examination (a–f) and EDS composition analysis (g) of the tribofilm on cast-iron flat lubricant by PAO4 + 0.50 wt% ZnS NPs at 100 °C.

A thin cross section of the wear scar was lifted using FIB and prepared for the STEM nanostructure analysis. Fig. 7a, b and d show the low magnification STEM images of the ZnS mixed 75–250 nm thick tribofilm formed on the wear scar. The ZnS tribofilm was likely to be polycrystalline, as evident by high magnification STEM images and corresponding FFT diffraction pattern according to Fig. 7c and f. The EDS elemental maps show Zn and S co-distribution on the FeO_*x*_ rich tribofilm (Fig. 7g). The relative element composition (by atomic%) of the tribofilm found to be Fe: 33%, O: 19%, Si: 2%, Zn: 26% and S: 20%. The EDS line scan in Fig. S3† clearly shows element distribution along tribofilm to the substrate. The Zn and S were relatively high in the tribofilm but quickly dropped when approaching the cast-iron substrate. The ZnS nanocrystals were mechanically mixed with the tribofilm as seen in the cross-sectional images and EDS elemental maps. In some areas, localized ZnS NPs aggregation (10–15 nm size) was observed (Fig. 7b and c).

## 4. Discussion

The ZnS NPs are believed to reduce friction and wear by effectively reducing direct contact of surface counterparts with the formation of a ZnS tribofilm as shown in Fig. 7. The low shear strength of ZnS help to minimize the asperities collision to reduce the friction similar to the ZnS coating.^27^ The superior antiwear performance of the ZnS NPs could also be partially due to the crystallinity of the tribofilm. Under contact stress, nanocrystals possibly underwent smearing and mechanical mixing with the tribofilm matrix. Mechanical mixing of the ZnS NPs provide additional crystallinity to the tribofilm.^28^

Moreover, nanocrystalline structures inherit higher mechanical strength compared to amorphous structures and yield minimal motion of dislocation.^29^ Spikes *et al.* showed the importance of the crystallinity and orderliness of the ZDDP tribofilm for friction and wear performance of the two-contact interfaces.^30^ Therefore, nanocrystalline additives such as ZnS NPs might provide an additional advantage over non-crystalline additives such as ZDDP.

Further improved friction and wear performance of ZnS NPs compared to the ZDDP at 150 °C could be due to the higher thermal stability of ZnS NPs, as shown in the TGA analysis. SEM wear scar morphology of ZDDP lubricated cast iron flat at 150 °C showed significant surface damages and formation of pits and cracks (Fig. S1†), despite the deposition of Zn, S, and P compound on wear track (Fig. S2†). In contrast, ZnS NPs lubricated oil did not show pits or crack as well as rich with Zn and S deposition as shown in EDS spectra in Fig. S2.†

Liu *et al.* report tribological properties of DDP-coated ZnS compared with the non-coated ZnS NPs, and the lubricant performance was ascribed to the formation of ZnS incorporated boundary film during the friction process.^17^ Libo *et al.* evaluated the polyethylene glycol monomethyl ether dithiophosphate modified ZnS NP (5–10 nm) in PEG 400 oil and observed improved antiwear and friction reduction properties.^16^ The authors observed the presence of Zn, S, and P on the wear scar using XPS analysis and thus confirmed the tribological properties were due to the comprehensive effect of nanoparticles and the surface modifier. The authors further described the formation of protective boundary film due to the high activity of nanoparticles, tribochemical reaction at high temperatures and pressure at the contact area in the lubrication process. Similarly, we also observed a Zn- and S-rich tribofilm from both the top surface and cross-sectional analysis. Shi *et al.* modified the ZnS NPs using Cyanex 302 (di-(2,4,4-trimethylpentyl)monothiophosphinic acid) and studied the tribological prophets in liquid paraffin.^15^ They also reported the formation of tribofilm composed of ZnS and Fe_2_O_3_ by tribochemical reaction. In addition, they observed minimal ZnS oxidation due to the organic modification. Shuang *et al.* concluded the presence of both ZnS and ZnO in the tribofilm.^21^ Authors proposed possible bond breaking between ZnS and organic modifier under the high temperature and pressure caused by friction, exposing naked and high surface energy sites of the NPs susceptible to oxidization. The elemental TEM cross-section analysis in Fig. 7 shows oxygen distribution throughout the tribofilm; thus, embedded ZnS oxidation to ZnO could not be avoided. Organic-modified ZnO nanoparticles are also reported as a potential additive to reduce friction and wear. Mariño *et al.* used oleic acid-coated ZnO nanoparticles (10 nm) as an additive to the PAO40 oil.^31^ Wear scar analysis using confocal Raman spectroscopy shows the ZnO, oleic acid, and iron oxides on the tribofilm. Wu *et al.* studied the oleic acid-coated ZnO nanoparticle (4 nm) as an additive to the PAO and diisooctyl sebacate base oils.^32^ They observed a strong zinc peak on the wear scar, confirming the NPs incorporated surface protective and lubricious layer leading to reduced friction and wear.

Does the dodecanethiol ligands contribute to the friction and wear performance? TGA data (Fig. 3e) confirmed the 40% organic contents in the ZnS NPs. Therefore, 0.5 wt% of ZnS NPs in the PAO oil should contains approximately 0.2 wt% of organic ligand. Fig. S4a and b† compared the friction and wear results of neat PAO, PAO + 0.15% dodecanethiol, PAO + 0.35% dodecanethiol with PAO + 0.5% ZnS NPs. The addition of dodecanethiol to the PAO oil eliminated the initial friction spick, but it did not affect the steady-state friction very much (similar to that of the neat PAO). The ligand reduced wear volume significantly, though not as effective as the dodecanethiol-modified ZnS NPs. The anti-wear behavior of dodecanethiol might be due to the adsorption of the sulfur-containing ligand onto the contact surfaces and the involvement of S containing compound in tribofilm formation.^11^ For understanding the wear mechanism of dodecanethiol, we had examined the worn surface of a cast-iron flat substrate lubricated by PAO4 + 0.15% dodecanethiol.^11^ The cross-section analysis using STEM/EDS revealed the formation of a sulfur-containing, iron oxide-based 100–200 nm thick tribofilm. This is likely to the adsorption of the electron-rich thiolate group to the positively charged metal surface, followed by chemical reactions with iron to produce iron sulfide in the tribofilm. The tribological performance of the unmodified ZnS NPs was not considered due to the precipitation of NPs in oil without the organic ligand support. Therefore, the organic ligand provides secondary surface protection in addition to facilitating the suspension and dispersion of the ZnS NPs.

Performance of ZnS NPs compared to the Ag NPs and Pd NPs: Fig. S5† shows the friction and wear performance of Ag NPs, Pd NPs compared to the ZnS NPs at 0.5 wt% in PAO4 base oil for 1000 m sliding under 100 N load at 100 °C. According to the data, the average COF of Ag NPs, Pd NPs, and ZnS NPs were 0.10, 0.08, and 0.09, respectively. Thus, ZnS performed well with an additional 10% friction reduction compared to the Ag NPs under similar surface modification and tribological conditions. The ZnS NPs outperformed in wear by 70% and 34% compared to the Ag NPs and Pd NPs, as shown in Fig. S5b.† The Ag NPs and Pd NPs are considered metallic NPs (oxidation state of Ag^0^ and Pd^0^) despite their minor oxidation nature at the interface with the organic layer. The Ag NPs found to be mechanically mixed into the tribofilm, forming a 100 nm thick wear protective film.^10^ In contrast, cross-section analysis of Pd NPs lubricated tribofilm shows two types of tribofilm, one with 1–2 μm thick, which is 1–2 orders of magnitude thicker than commonly observed tribofilm in the literature. Additionally, PdNP-induced tribofilm was dominated by Pd/S compounds with just a little iron oxide filling.^11^ However, oxidation status of Zn in ZnS NPs is +2, since electron filled 3d^10^ shell do not participate in the chemical reaction.^33^ Therefore, in addition to the mechanical mixing to the tribofilm as ZnS NPs, ZnS can undergo tribochemical reactions to form ZnS-rich tribofilm. The contact stress at the sliding interface and asperity collision could lead to (a) NPs aggregation and deposition onto the contact surfaces. (b) Detachment of the organic ligand from the ZnS NPs interface.^21^ (c) ZnS on the detached NPs surface might further oxidizer to ZnO or act as a cation supply to the tribochemical reaction to trigger tribofilm formation. But, ZDDP tribofilm formation mechanism is quite different.^13^ Under high temperature and pressure at the contact interface, ZDDP initially breakdown to release Zn^2+^ and phosphate ions and then reacts with oxygen and the iron surface to grow a tribofilm on the contact area. This thermal degradation of ZDDP leads to zinc phosphate, thiols, and olefins. The superior wear protection mechanism of the ZnS NPs compared to the ZDDP, AgNPs, and PdNPs might be due to the dual antiwear mechanisms. (a) Nano-size particles are embedded to tribofilm and act as cushion for asperity collision and (b) the cationic nature of the decomposed ZnS NPs to enhance tribochemical interactions during tribofilm formation. Preliminary screening of 1% ZnS NPs in 5W-20 and 0W-20 commercial engine oils suggested the necessity of dedicated formulation to optimize the antiwear performance of ZnS NPs.

## 5. Conclusions

Dodecanethiol-modified ZnS NPs were synthesized using a non-aqueous wet chemical route and scaled up to obtain gram quantities of nanomaterials. TEM analysis shows the narrow and homogeneous size distribution of ZnS NPs. The dodecanethiol-modified ZnS NPs could be easily dispersed and suspended in a PAO4 base oil without any dispersant and have good optical transparency. The effective diameter of the 0.5 wt% ZnS NPs in PAO4 oil found to be 10 ± 1 nm using dynamic light scattering technique. Tribological bench tests were conducted at room temperature, 100 and 150 °C under reciprocating sliding at 100 N load for 1000 m. The ZnS NPs performed very well when added into the PAO4 oil at 0.5 or 1.0 wt%, providing effective scuffing prevention and excellent friction and wear protection, outperforming a commercial ZDDP. The friction reduction of 13–18% was achieved at 100 °C compared to the ZDDP. Additional wear reduction over ZDDP was 47%, 65%, and 67% at 23, 100, and 150 °C, respectively. Top and cross-sectional worn surface analyses indicated the formation of a Zn and sulfur-rich polycrystalline 75–250 nm thick protective tribofilm on the contact area, which is believed to be responsible for friction and wear reduction. The ZnS NPs would be a potential high-performance antiwear additive for broad lubrication applications. Dedicated oil formulation is required to gain a best performance of the ZnS NPs in commercial engine oil.

## Conflicts of interest

There are no conflicts of interest to declare.

## Supplementary Material

RA-013-D2RA07295E-s001
