# Supplementary material for: Organic-modified ZnS nanoparticles as a high-performance lubricant additive
Source: RSC Adv. 2023 Mar 1;13(10):7009–19. doi: 10.1039/d2ra07295e (PMC9977461; doi:10.1039/d2ra07295e)
Supplement: RA-013-D2RA07295E-s001 [file RA-013-D2RA07295E-s001.pdf]

Electronic Supplementary Material (ESI) for RSC Advances.
This journal is © The Royal Society of Chemistry 2023

Supporting information

Organic Modified ZnS Nanoparticles as High-Performance Lubricant Additive

Chanaka Kumara,[1,*] Beth Armstrong,[1] Inwoong Lyo,[2] Hong Wook Lee,[2] Jun Qu[1,*]

[1]Materials Science and Technology Division, Oak Ridge National Laboratory, Oak Ridge, TN, 37830, USA.
[2] Institute of Advanced Technology Development, Hyundai Motor Corporation, Republic of Korea

Co-corresponding Authors

*kumarack@ornl.gov and qujn@ornl.gov

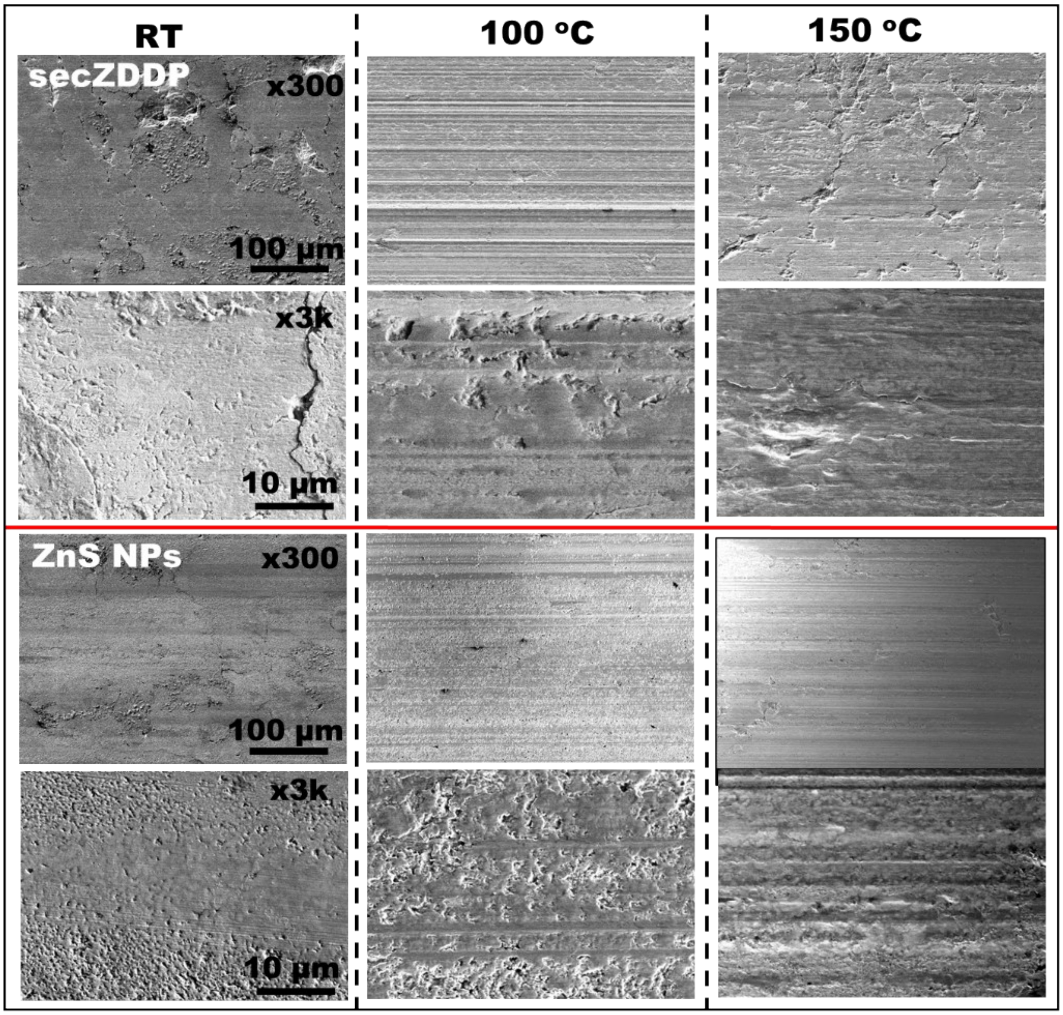

Figure S1. Wear scars morphology comparison of the cast-iron flat lubricated by PAO+0.8wt% secZDDP and PAO+1wt% ZnS NPs at RT (23 ^{o}C), 100 and 150 ^{o}C.

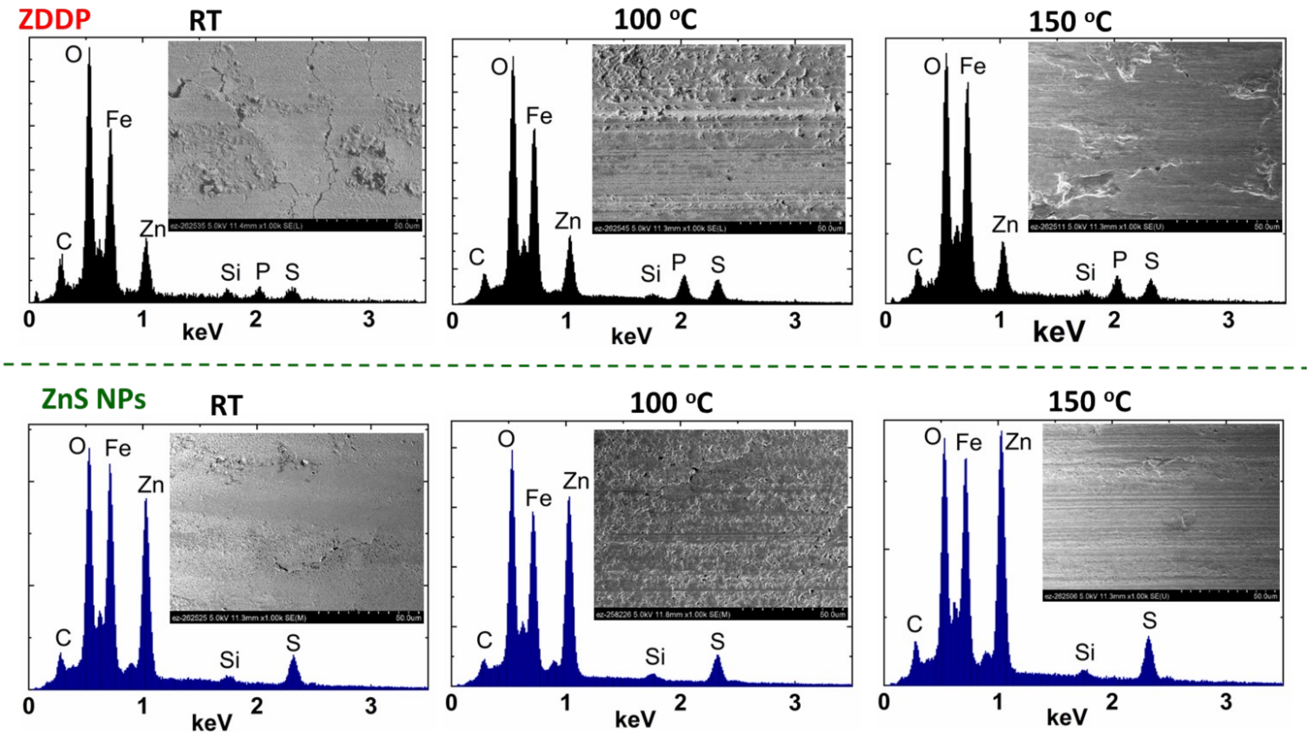

Figure S2. Surface morphology and EDS spectra comparison of the cast-iron flat lubricated by PAO+0.8wt% secZDDP and PAO+1wt% ZnS NPs at RT (23 °C), 100 and 150 °C.

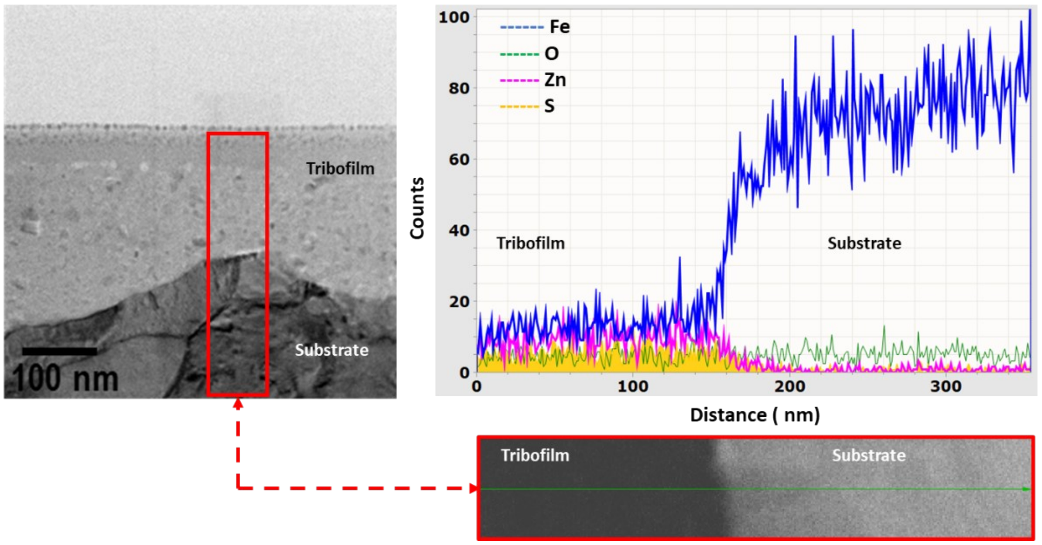

Figure S3. EDS line scan through the red reference zone as marked on the STEM image showing elemental distribution.

(a) (b)

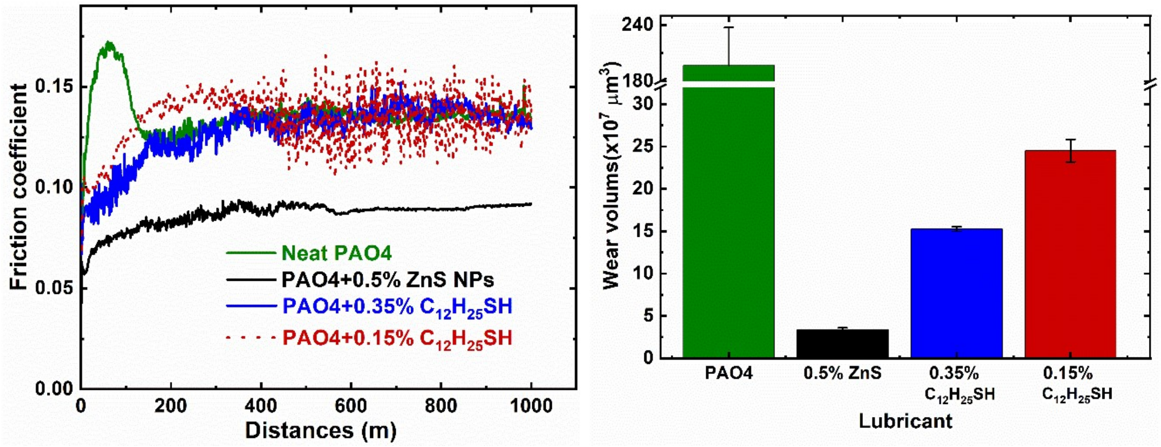

Figure S4. Friction (a) and wear (b)performance of the organic modified ZnS NPs compared with using the organic ligand alone in the neat base oil.[1]

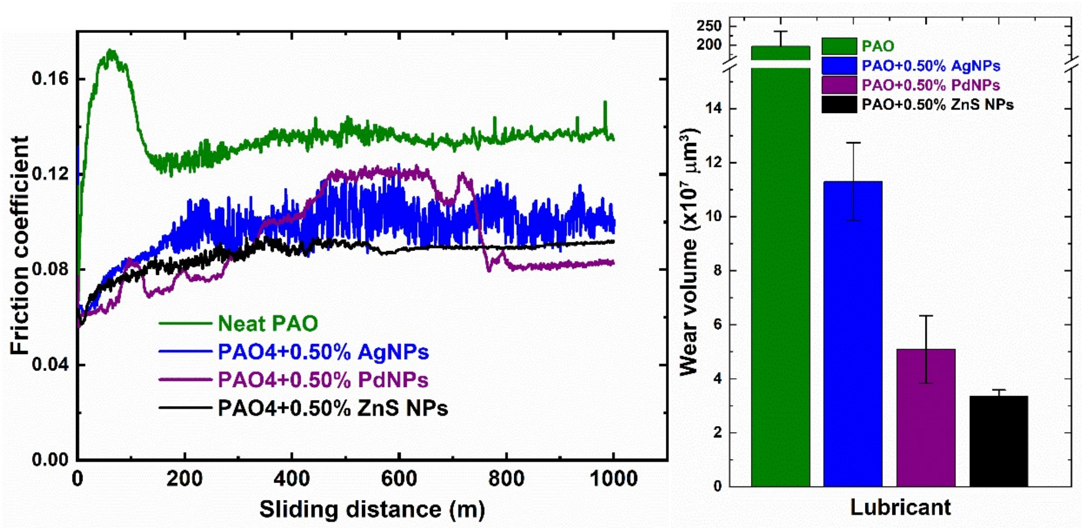

Figure S5. Friction (a) and wear (b) performance of the organic modified AgNPs, Pd NPs and ZnS NPs in PAO4 base oil for 1000 m sliding under 100 N load at 100 ^{o}C. [1]

Referance

(1) Kumara, C.; Leonard, D. N.; Meyer, H. M.; Luo, H.; Armstrong, B. L.; Qu, J. Palladium Nanoparticle-Enabled Ultrathick Tribofilm with Unique Composition. *ACS Applied Materials & Interfaces* **2018,** *10* (37), 31804-31812.
